# Resting-state functional connectivity identifies individuals and predicts age in 8-to-26-month-olds

**DOI:** 10.1016/j.dcn.2022.101123

**Published:** 2022-06-15

**Authors:** Omid Kardan, Sydney Kaplan, Muriah D. Wheelock, Eric Feczko, Trevor K.M. Day, Óscar Miranda-Domínguez, Dominique Meyer, Adam T. Eggebrecht, Lucille A. Moore, Sooyeon Sung, Taylor A. Chamberlain, Eric Earl, Kathy Snider, Alice Graham, Marc G. Berman, Kamil Uğurbil, Essa Yacoub, Jed T. Elison, Christopher D. Smyser, Damien A. Fair, Monica D. Rosenberg

**Affiliations:** a University of Chicago, USA; b Washington University in St. Louis School of Medicine, USA; c University of Minnesota, USA; d Oregon Health & Science University, USA

**Keywords:** Functional connectivity, FMRI, Reliability, Development, Machine learning, Age prediction

## Abstract

Resting-state functional connectivity (rsFC) measured with fMRI has been used to characterize functional brain maturation in typically and atypically developing children and adults. However, its reliability and utility for predicting development in infants and toddlers is less well understood. Here, we use fMRI data from the Baby Connectome Project study to measure the reliability and uniqueness of rsFC in infants and toddlers and predict age in this sample (8-to-26 months old; *n* = 170). We observed medium reliability for within-session infant rsFC in our sample, and found that individual infant and toddler’s connectomes were sufficiently distinct for successful functional connectome fingerprinting. Next, we trained and tested support vector regression models to predict age-at-scan with rsFC. Models successfully predicted novel infants’ age within ± 3.6 months error and a prediction R^2^ = .51. To characterize the anatomy of predictive networks, we grouped connections into 11 infant-specific resting-state functional networks defined in a data-driven manner. We found that connections between regions of the same network—i.e. within-network connections—predicted age significantly better than between-network connections. Looking ahead, these findings can help characterize changes in functional brain organization in infancy and toddlerhood and inform work predicting developmental outcome measures in this age range.

## 1 Introduction

The first two years of life are characterized by rapid development of behavioral capabilities and dramatic changes in brain structure and function (Benson, 2020, Silbereis et al., 2016, Almli et al., 2007), including changes in functional network organization (Gao et al., 2015a, Gao et al., 2015b, Cao et al., 2017). Resting-state functional connectivity (rsFC) measured with functional MRI offers one way to measure functional brain organization as well as changes in this organization over time. In particular, changes in rsFC patterns over time have been shown to relate to development, indexed with chronological age, in childhood and adulthood (Nielsen et al., 2019, Dosenbach et al., 2010), as well as the first year of life (Pruett et al., 2015). However, rsFC’s reliability and utility in tracking development in the second year of life—a period marked by many developmental milestones (Lewis and Ramsay, 2004, Hayne et al., 1997, Courage and Howe, 2002) but one of understudied functional brain architecture (Edde et al., 2021)—is unclear. In the current work, we evaluate the reliability of rsFC patterns in infants between the ages of 8–26 months and characterize its relationship to chronological age.

Functional connectivity is typically measured by calculating the statistical dependence (e.g., correlation) between two brain regions’ blood-oxygen-level-dependent (BOLD) signal time series. Doing so for all pairs of brain regions in a whole-brain parcellation scheme, or brain atlas, results in a functional connectivity matrix, or connectome, that reflects aspects of an individual’s functional brain organization (Van Den Heuvel and Pol, 2010, Hlinka et al., 2011, Kelly et al., 2012, Hermundstad et al., 2013). In adults, these rsFC *patterns* are relatively stable over time and unique across individuals (Tozzi et al., 2020), although the univariate reliability of *single* functional connections is poor (Noble et al., 2019) as indicated by low intra-class correlation (ICC) for single connections (~.29). Individuals’ overall functional connectivity patterns individuate and stabilize across childhood and adolescence and delays in connectome individuation and stabilization are observed in psychiatric disorders (Kaufmann et al., 2017). This suggests that these connectivity changes are relevant for neurocognitive development. But, the degree to which individuals’ unique functional connectome “fingerprints” (Miranda-Dominguez et al., 2014, Finn et al., 2015) are apparent in infancy is unclear. Although recent work has demonstrated medium to large intra-session reliability of resting-state functional connectivity in newborn infants (Wang et al., 2021a), other work suggests that functional connectome identifiability—a proxy for connectome reliability and uniqueness—is poor in infancy (Dufford et al., 2021)

In addition to showing reliability over short time scales, adults’ rsFC patterns also show high long-term inter-session *stability*, meaning different scanning sessions months apart would yield similar rsFC matrices for an adult individual (Horien et al., 2019; see Miranda-Dominguez et al., 2018; Jalbrzikowski et al., 2019 for adolescents). In infancy and toddlerhood, however, large developmental changes in rsFC could occur in short time scales such as months or even weeks, and therefore high long-term stability would be surprising (e.g., see Dufford et al., 2021). Rather than long-term stability, the question in the current study is whether the rsFC changes across development in infancy and toddlerhood are systematic and could be informative about the brain maturation. To understand rsFC’s potential utility for indexing brain maturation, it is important to understand the reliability of infant and toddler’s functional connectomes’ relationship with development. Work has shown the functional connectome’s relationship with categorical age (i.e., 6-month-old vs. 12-month-old infants, Pruett et al., 2015) and socioeconomic status (Gao et al., 2015a, Gao et al., 2015b) in the first year of life. However, its utility to predict continuous brain development indexed as chronological age in months during the late infancy and toddlerhood is not known. Predicting age based on rsFC enables us to understand systematic functional architectural changes during the brain maturation process in these early years, which in turn could help studies aiming to find brain indices of typical and atypical neuro-development of other phenotypes in cognitive, language, motor, and other domains.

In the current study, we used a relatively large, high-quality MRI sample to investigate whether resting-state functional connectivity observed between 8 and 26 months is reliable within a scan session, distinct between individuals, and predictive of age in months. To do so, we first quantified the consistency of infants’ and toddlers’ rsFC patterns within a single scan session. We then assessed rsFC distinctiveness by comparing within-participant FC pattern similarity to between-participant FC pattern similarity (a “fingerprinting” analysis; Miranda-Dominguez et al., 2014; Finn et al., 2015). We next quantified the degree to which rsFC predicted age, asking whether it explained additional variance in brain maturity above and beyond brain volume. Finally, we compared the degree to which different functional networks predicted age to characterize functional network development in this cohort.

## 2 Methods

### 2.1 MRI data

Data from 259 MRI sessions collected when infants and toddlers were between 8 and 26 months old from the University of Minnesota site of the publicly available Baby Connectome Project (BCP; Howell et al., 2019) were used in this investigation. Study samples were preprocessed and visually assessed for quality. After exclusions (see *Data exclusion* section below), 170 scan sessions remained for analysis (mean age = 15.7 months, SD = 5.2; range = 8–26 months). Included sessions were collected from 112 unique participants (52 female). When a participant had multiple included sessions, only one session was randomly sampled within each analysis iteration. Sessions from the same participants were collected at least 3 months apart. The BCP Study was approved by the University of Minnesota and University of North Carolina Institutional Review Boards and informed consent was acquired from the parents of all participants.

In each MRI session, T1-weighted (TR=2400 ms, TE=2.22 ms, 0.8 mm isotropic), T2-weighted (TR=3200 ms, TE=563 ms, 0.8 mm isotropic), spin echo fieldmaps (SEFM) (TR=8000 ms, TE=66 ms, 2 mm isotropic, MB=1), and resting-state fMRI (TR=800 ms, TE=37 ms, 2 mm isotropic, MB=8) data were collected from participants on a Siemens 3 T Prisma scanner with a 32-channel head coil. Resting-state fMRI data were collected in both the Anterior→Posterior (AP) and Posterior→Anterior (PA) phase encoding directions. Each BOLD run consisted of 420 frames (5.6 min) with a minimum of two (11.2 min) and maximum of four runs (22.4 min) collected per scanning session. A subset of early scans (*n* = 68 sessions) was collected with a 720-ms TR. All scans were performed during natural sleep without the use of sedating medications, following procedures outlined in Howell et al. (2019).

### 2.2 Functional MRI data processing

Functional data processing steps were similar to those described in Feczko et al. (2021). Structural MRI data undergo an HCP-style processing (see: Glasser et al., 2013, Feczko et al., 2021), where the structural data undergo ANTS N4 bias correction, ANTS denoising, T1/T2 distortion correction/registration, and finally ANTS SyN algorithm deformation alignment to the infant MNI template. In addition, we performed a segmentation using templates derived for 8–26 months via Joint Label fusion, and produced a refined brain mask from this step. The mask and segmentation here were substituted within the freesurfer (Fischl, 2012) pipeline and used to refine the white matter segmentation, and guide the freesurfer surface delineation. The data were then converted to a CIFTI-template via a spherical registration from the native surfaces to the fsaverage LR32k.

For fMRI preprocessing, a scout image was selected from the resting-state fMRI timeseries. The scout was used to perform distortion correction via spin-echo field maps, served as the reference for motion-correction via rigid-body realignment (Feczko et al., 2021), and registered to the native T1. These steps were combined in a single resampling with the MNI template transformation from the previous step, such that all fMRI frames were registered to the infant MNI template. Mode 1000 normalization was performed, so that 10 units of BOLD correspond to a 1% signal change.

Standard connectivity processing steps were then applied beginning with demeaning/detrending across time. Denoising was performed using a general linear model with regressors including signal and motion variables. Signal regressors include mean CIFTI grey-ordinate timeseries, JLF-defined white matter, and JLF-defined cerebrospinal fluid (CSF). Motion regressors include volume-based translational and rotational components and their 24 parameter Volterra expansion. Framewise displacement (FD) was defined as the squared sum of the motion vectors provided by the frame alignment during fMRI pre-processing. As in adults, infant respiration can lead to perturbations in the B_0_ field, which, unlike spontaneous isolated head movements, do not result in BOLD signal disruption (Fair et al., 2020). This factitious head motion should not be taken as an indicator of degraded image quality. Following the methods of Kaplan et al. (2021) which showed that applying an age-specific respiratory notch filter (0.28–0.48 Hz) to the FD traces and motion parameter estimates in this dataset successfully mitigates the effects of respiratory motion, we removed the respiratory apparent head motion and retained more of the data compared to motion censoring with no FD filtering (Kaplan et al., 2021). Frames were censored during demeaning/detrending if their FD value exceeded 0.3 mm. Consequently, the denoised beta values only included the remaining low motion frames, while keeping a sufficient number of frames to demean/detrend. Bandpass filtering was applied using a second-order Butterworth filter (0.008–0.09 Hz). To preserve the temporal sequence and avoid aliasing caused by missing timepoints during bandpass filtering, interpolation was used to replace missing frames, and residuals were acquired from the denoising general linear model. Parcellated timeseries were generated by averaging the voxel-wise time-series within each parcel using a predefined 333-node cortical atlas (Gordon et al., 2016) which defined parcels using rsFC boundary maps calculated by “watershed by flooding” algorithm in a sample of young adults (see Gordon et al., 2016).” Frames with FD values exceeding 0.2 mm were excluded from any connectivity calculations.

### 2.3 Data exclusion

Frames with > 0.2 mm FD or outlier frames whose across-voxel standard deviation was more than 3 median absolute deviations from the median of all frames were removed from the BOLD timeseries prior to functional connectivity analysis. Scan sessions with fewer than 600 total frames (i.e., 7.2–8 min of data) across all runs were excluded, resulting in 231 sessions. Visual quality control was performed on T1-weighted, T2-weighted, and BOLD images (at least 2 independent raters per image) with the Swipes for Science platform (https://swipesforscience.org). Sessions with less than a 75% aggregated passing rate across images and raters on either anatomical or functional images were excluded, resulting in our final sample of 170 sessions.

### 2.4 Functional connectivity matrix construction

Preprocessed 333 Gordon parcellated cortical BOLD timeseries (Gordon et al., 2016) were truncated to their first *k* frames (*k* = 5, 10, 15,., 450 in the fingerprinting analysis and *k* = 300 in the age prediction analysis, described below) from the AP and PA scans separately. A Pearson correlation matrix was constructed for each session by pairwise correlating the 333 truncated time-series, resulting in 55,278 unique functional connections in each FC matrix.

### 2.5 Functional connectome fingerprinting

**Fig. 1:**
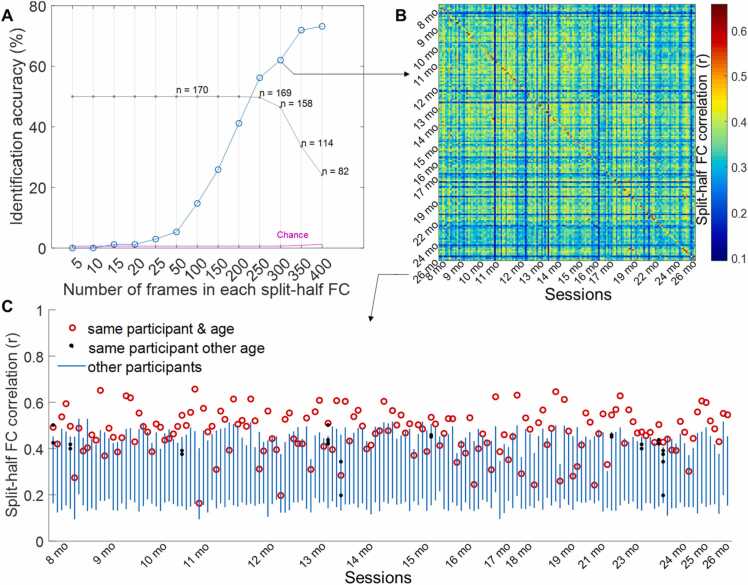
A: Functional connectivity fingerprinting identification percent accuracy (in blue) as a function of number of frames (*k*) included in each split-half connectivity matrix generated from AP and PA data. The grey curve reflects data retention at a given *k*. For example, *n* = 158 at *k* = 300 indicates that, of the 170 sessions in the sample with at least 600 frames after motion and outlier censoring, 158 had at least 300 frames in each AP or PA direction and were thus included in the analysis. The pink line shows chance % (i.e., 100 divided by *n*). B: Matrix plot where sessions, ordered by age, are rows/columns and cells reflect the correlation between sessions’ split-half functional connectivity patterns at *k* = 300. **C:** When 300 frames were used to generate each split-half matrix, overall identification accuracy was 62%. That is, 62% of sessions’ AP matrices are more correlated with their corresponding PA matrix than with the PA or AP matrix from any other participants’ session. Blue lines show the range of all between-participant correlations—of any ages— for each session. Sessions are ordered along the *x*-axis by age.

As one way to assess the reliability of subject-level FC in our sample, we performed a “fingerprinting” analysis (Miranda-Dominguez et al., 2014, Finn et al., 2015). This analysis tests the degree to which individuals’ FC patterns are stable over time and unique from a group. To this end, we constructed pairs of FC matrices using the 333 parcels for each session (*n* = 170) using the first *k* TRs from AP and PA resting-state runs. We then computed the spatial correlation between these AP and PA FC matrices for all pairs of sessions. Correct identification was achieved if, at a given *k*, the correlation between the AP and PA matrices from the same participant was larger than the correlation between the AP matrix from that participant with the PA matrices of all other sessions of other participants and the correlation between the PA matrix from that participant with the AP matrices of all other sessions of other participants (excluding other sessions from the same participant). AP and PA runs were run consecutively and half of the sessions (87 out of 170) only had two runs of fMRI (one AP and one PA). Therefore, the fingerprinting was done by correlating AP and PA runs as opposed to two AP runs and two PA runs, which would have excluded the sessions with fewer than 4 good quality runs (two AP and to PA). Overall identification accuracy for a given *k* was calculated as the percent of successful identifications across all sessions with at least *k* frames of data in both AP and PA runs, excluding sessions from the same participant if they had multiple sessions (see *Results* section Fig. 1). Please note that for simplicity, we refer to the FC from the AP or PA run within a session as split-half FC in Fig. 1 A, and refer to FC similarity of the two runs (one AP and one PA) within a session as split-half correlation in Fig. 1B of the *Results* section. *Supplementary section 1* contains a few examples of split-half matrices plotted side-by-side for comparison.

### 2.6 Test-retest reliability of single edges

Intra-class correlation (ICC) for each edge in the FC matrices were calculated as two-way random single score absolute ICC (Shrout and Fleiss, 1979, McGraw and Wong, 1996) for two runs in the same session using custom MATLAB code based on Thomas Zoeller (2022) implementation (https://www.mathworks.com/matlabcentral/fileexchange/26885-intraclass-correlation-coefficient-with-confidence-intervals). The measure compares the variance due to participants compared to variance due to the two runs and noise, with higher ICC value for an edge indicating more within-session reliability of that connection between two runs of a participant. ICC was averaged over all edges to assess the edge-wise reliability in the sample.

### 2.7 Age prediction

Preprocessed 333 Gordon parcellated timeseries were truncated to their first 300 AP and first 300 PA frames (or the closest balance between AP and PA possible) for a total of 600 frames in all 170 sessions. For 12 of the 170 sessions, there were fewer than 300 TRs in one acquisition direction (maximum difference = 240 AP and 360 PA TRs). A Pearson correlation matrix was then calculated from these 600 TRs and Fisher *z*-transformed (tanh^−1^) to normalize the connectivity values.

Support vector machine regression (SVR) with a linear kernel function (Christianini and Shawe-Taylor, 2000) and 10-fold cross-validation was used to predict infants’ and toddlers’ age in months from their resting-state FC pattern. An extension of support vector machine (SVM) learning for continuous prediction, SVR (Vapnik, 1995), uses a training set of observations with known ages and extracts the multivariate relationship between predictors (i.e., functional connections, or edges) and age as the continuous variable of interest. Predicted age values were aggregated across the 10 folds and assessed with measures of model performance: Pearson correlation between predicted and true age (*r*) and prediction R^2^ coefficient of determination (Alexander et al., 2015) (see *Supplementary section 2* for more detail).

In our final sample of 170 MRI sessions, some sessions were collected from the same participants at two different ages. To avoid potential issues of data clustering, we randomly selected one session per participant (*n* = 112) 500 times and applied 10-fold SVR training and cross-validation to predict age. This approach resulted in a distribution of 500 *r* and prediction R^2^ estimates, which were compared to null distributions to assess significance. Null distributions were generated by applying 10-fold SVR to data from the same 112 sessions used to train and test the true models after permuting age values.

### 2.8 Nuisance variables

In addition to measuring *r* and prediction R^2^, we assessed the partial correlation between true and predicted age (partial *r*), adjusted for nuisance variables in each iteration. Nuisance variables included mean remaining frame-to-frame head displacement in the session after motion censoring and outlier frame removal (FD), average visual quality control rating for functional and anatomical images for the session (QC ratings), and a dummy variable encoding whether the TR for the session was 0.72 s or 0.8 s (TR).

### 2.9 Predicting age above and beyond brain volume

To test whether FC patterns predict chronological age above and beyond overall brain volume, we compared the variance in age explained by total brain volume alone to the variance explained by total brain volume and FC together. This provides an estimate of how changes in the brain’s *functional* architecture in this age range are coupled with brain maturity and predict age. This estimate is conservative in that all FC-based variance in age shared with brain volume is attributed to the brain volume model.

To this end, we estimated total brain volume at each session using *recon-all* from *Freesurfer* (values in mm^3^ were taken directly from each subject’s *aseg.stats* file). We then regressed age in each of the 500 resamples of 112 participants on nuisance variables (Model 1: Null), as well as nuisance variables plus brain volume and brain volume squared (Model 2: Brain volume). We calculated explained variance (adjusted R^2^) in each regression, resulting in a distribution of adjusted R^2^ values for each of Model 1 and Model 2. In Model 3: resting-state FC, SVR-predicted age (i.e., age fit score for the test 10-fold partitions) was then introduced in the linear regression model to calculate the additional explained variance uniquely contributed to age prediction from FC, above and beyond brain volume and nuisance variables. The ΔR^2^ in each of the regressions in the 500 data resamplings were calculated and its distribution was compared to 0 (i.e., the. Null hypothesis of no additional explained variance between Model 2 and Model 3).

### 2.10 Investigating predictive power of within-network vs. between-network edges

Reducing the feature space in connectivity-based predictive models can be performed in a data-driven or hypothesis-driven manner. Data-driven feature selection is optimal for reducing dimensionality without losing model generalizability but comes with the trade-off of potentially less interpretable results. Hypothesis-driven feature selection may penalize predictive power but can produce better theoretical interpretations. To explore the functional anatomy of the functional connections that predict age in our sample, we divided edges into two groups based on whether their nodes belonged to the same functional network or different networks. We used functional network definitions generated in the BCP Study sample itself and in an adult sample (Gordon et al., 2016).

To define networks in the BCP Study sample, Infomap (Rosvall and Bergstrom, 2008) was applied to 8-to-26-months old data to generate BCP-specific networks using previously published methods (Wheelock et al., 2019, Eggebrecht et al., 2017). In brief, FC data from 94 BCP individuals (a single session per participant) were used to generate a group-average 333 × 333 connectome using the Gordon parcels (Gordon et al., 2016). The included subjects had a Mean age = 16.55 months (range 9–24 months) and had a minimum of 5 min of data (Mean=16.02 min, range 5.02–29.27 min) after motion censoring at FD threshold of 0.2 mm. To estimate pediatric-specific brain networks, the group average BCP connectome was thresholded across a range of edge densities (1–10%) and binarized as input into the Infomap community detection algorithm (Rosvall and Bergstrom, 2008). Although the Gordon 333 parcel boundaries were identical to those used in adults, the parcels were assigned to 11 pediatric functional networks rather than the (Gordon et al., 2016) networks (Fig. S2). These surface-based pediatric functional networks resemble previously published infant and toddler networks derived using volumetric, spherical regions of interest (Eggebrecht et al., 2017).

Importantly, the 11 infant/toddler networks were identified using FC data from a representative age range across all sessions and no within-dataset age-related variance is used in detection and assignment of communities. This is important because the 94 sessions used in the Infomap analysis were also included in the 170 sessions used in the age prediction analyses using within-network and between-network edges. (In other words, defining which edges constitute a community is not part of the cross-validation procedure used to compare the age-predictive power of within-network vs. between-network edges).

## 3 Results

### 3.1 Functional connectome fingerprinting and reliability

Infants’ and toddlers’ rsFC patterns were more similar within participants than across participants with medium level of identifiability (60%−70%). Specifically, split-half correlation analysis (one half being the AP run and the other half being the PA run, see Methods) showed that resting-state functional connectomes constructed from only 300 frames (3.6–4 min depending on the TR) in each half successfully distinguished the identity of the participant, with 62.0% accuracy across all available sample (*n* = 158 sessions with at least 2 × 300 frames, chance = 1/158 = 0.63%; Fig. 1A). As expected, identification accuracy increased as more frames were used to calculate the split-half FC matrices. Accuracy plateaued at 2 × 350 frames to 71.9% with a peak of 73.2% at 2 × 400 frames, although identification was numerically above chance with as few as 20 frames per half.^1^ Additionally, at 2 × 300 frames, we found medium reliability for within-session rsFC (mean correlation between runs from the same session: *r* = 0.470, *SD* = 0.098; Fig. 1B-C). The test-retest reliability for single edges (i.e., individual connections), however, was low as shown by low average edge-level intra-class correlations (mean ICC = 0.20, SD = 0.13). This follows previous reports on low univariate reliability of single functional connections for infants (Dufford et al., 2021, Wang et al., 2021a) and adults (Noble et al., 2019).

Notably, functional connectome identification accuracy was not driven by only older participants. Within-participant FC similarity did not increase with age (correlation between age and within-participant FC similarity: *r* = 0.064, p = .421; red circles in Fig. 1C are not higher for older sessions; sessions are ordered by age along the x axis). A participant’s distinctiveness from the null distribution also did not significantly increase with age (β = 0.05, p = .108 from logistic regression of identifiability on age; blue bars in Fig. 1 C). Thus, FC observed between 8 and 26 months contains signal that can reliably identify individuals.

Finally, analyses suggest that FC similarity is not strongly driven by age in this sample (Fig. 1B). That is, the correlation between the FC similarity matrix in Fig. 1B and a corresponding age difference matrix (in which values reflect the difference in age between two sessions) was low albeit statistically significant (*r* = −0.097, p = .001 from permuting matrix elements 1000 times). In other words, rsFC pairs from more similar ages are slightly more similar than rsFC pairs from more distant ages across participants.

### 3.2 Predicting age from functional connectivity

#### 3.2.1 Functional connectivity patterns predict age in months

**Fig. 2:**
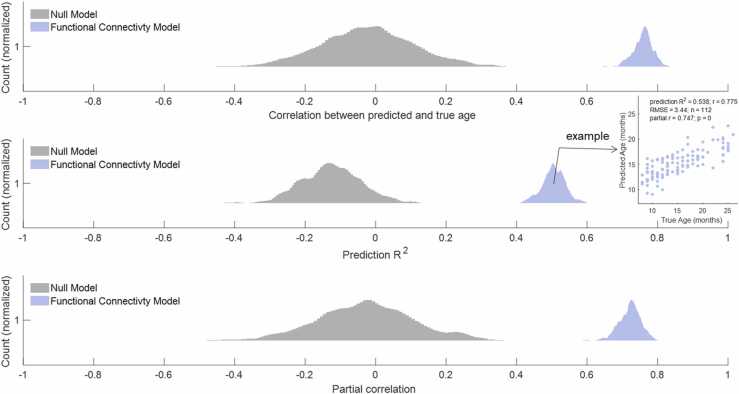
Distributions of age-prediction model performance metrics. **Top:** Pearson correlation between predicted age and true age. **Middle:** Prediction R^2^ estimated with mean squared error (MSE). **Bottom:** Partial correlation of predicted and true age adjusted for nuisance measures (remaining head motion, manual quality ratings, TR).

Functional connectivity patterns successfully predicted infants’ and toddlers’ age in months when assessed with all measures of prediction performance (Fig. 2). Pearson’s correlations between true and predicted age were high (median *r* = 0.762, *p*_z-test_ <0.0001, *p*_perm_ < 1/500), with relatively precise predictions (median RMSE = 3.59 months) resulting in high prediction R^2^ (median prediction R^2^ =.506, *p*_z-test_ <0.0001, *p*_perm_ < 1/500). Results were consistent after adjusting for nuisance variables (FD, QC ratings, and TR; median partial *r* = 0.724, *p*_z-test_ <0.0001, *p*_perm_ < 1/500). Thus, continuous age in months is well-captured by functional connectivity in this sample of 8-to-26-month-olds.

#### 3.2.2 rsFC predicts age beyond brain volume

**Fig. 3:**
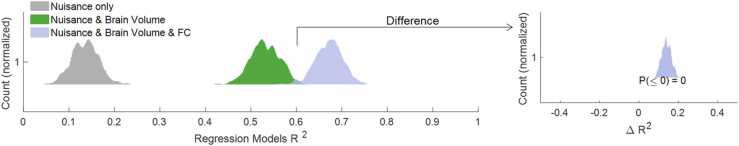
Distributions of R^2^ for age-prediction models based on nuisance variables (mean frame-to-frame head motion post-censoring, visual quality control rating, number of TRs; grey), with the addition of brain volume and brain volume squared (green), and the addition of rsFC-predicted age (blue). The addition of rsFC-predicted age significantly increased the explained variance in age (distribution of change in R^2^ shown in the right panel).

We next asked if rsFC patterns carry reliable signal about chronological age above and beyond anatomical measures of brain volume. As shown in Fig. 3, rsFC patterns contributed to age prediction above and beyond brain volume measures (median ΔR^2^ =.139, *p*_perm_ < 1/500).

### 3.3 Functional anatomy of predictive connections and their relationship with age

#### 3.3.1 Adult and infant functional network assignments

**Fig. 4:**
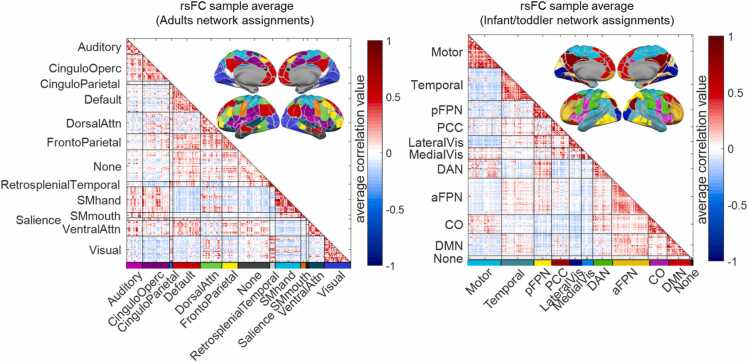
Functional connectivity averaged over our sample of 8-to-26-month-olds. Nodes are arranged according to adult-defined canonical functional networks from (Gordon et al., 2016) (left) or BCP-specified functional networks (right). As expected, the BCP infant/toddler network assignments better capture the clusters of coordinated activity in infants as indicated by the better aggregation of stronger elements along the diagonal. Acronyms for infant networks names: DAN = Dorsal Attention Network; PCC = Posterior Cingulate Cortex; CO = Cingulo-Opercular; DMN = Default Mode Network; aFPN/pFPN = anterior/posterior Fronto-Parietal Network.

The average rsFC pattern across all 170 sessions is shown in Fig. 4, arranged in two ways. Here, we find that the functional “adjacency” of the nodes is better captured with the 11 infant/toddler network assignments rather than when nodes are arranged according to 12 canonical adult functional networks. This is indicated by the better aggregation of stronger elements along the diagonal in the right panel (mean within-network functional connectivity in infant/toddler networks arrangement *r* = 0.261, *SD* = 0.165) compared to left (mean within-network functional connectivity in adult networks arrangement *r* = 0.201, *SD* = 0.187; Cohen’s *d* = 0.241 between the two means). Since the canonical adult networks do not well-capture clusters of coordinated activity in our sample as well as the infant/toddler networks, we proceeded with the infant/toddler network assignments to explore the functional anatomy of age-predictive edges in the better fit community space.

#### 3.3.2 Within-network connections better predict age than between-network connections

**Fig. 5:**
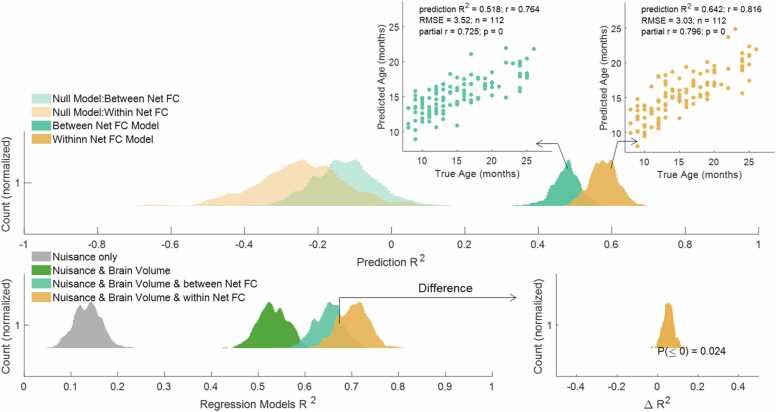
**Top:** Performance of the rsFC age-prediction models using only within-network connections (orange) or between-network connections (teal). See Supplementary Fig. S3 for feature maps of the example models. **Bottom:** The within-network rsFC model significantly outperformed the between-network rsFC model when predicting age, above and beyond the brain volume measures model. The bottom right plot shows the distribution of difference in R^2^ between the additional variance in age (above and beyond nuisance variables and brain volume) explained by within-network and between-network connections.

After showing the relatively large predictive power of whole-cortex functional connectivity patterns for age, we investigated whether a subset of all 55,278 edges achieve similar performance. Specifically, more functionally similar vs. distinct edges (irrespective of the anatomical distance of the nodes) were separated into two models, one including only the 6017 within-network edges and the other including the remaining 49,261 edges—those crossing network boundaries. The within-network model performed better than the between-network FC model in terms of precision (within-network: median RMSE = 3.31 months and median prediction R^2^ =.579 vs. between-network: median RMSE = 3.69 months and median prediction R^2^ =.478). Prediction R^2^ of the within-network model was significantly greater than that of the between-network FC model (*p* = .008; Fig. 5, top panel). Furthermore, the amount of unique explained variance above and beyond nuisance variables and brain volume in the within-network regressions was significantly larger than that of the between-network regressions (median ΔR^2^ =.053, *p* = .024, between the two regression models’ R^2^; Fig. 5 bottom panels).

#### 3.3.3 Predictive power of functional networks

**Fig. 6:**
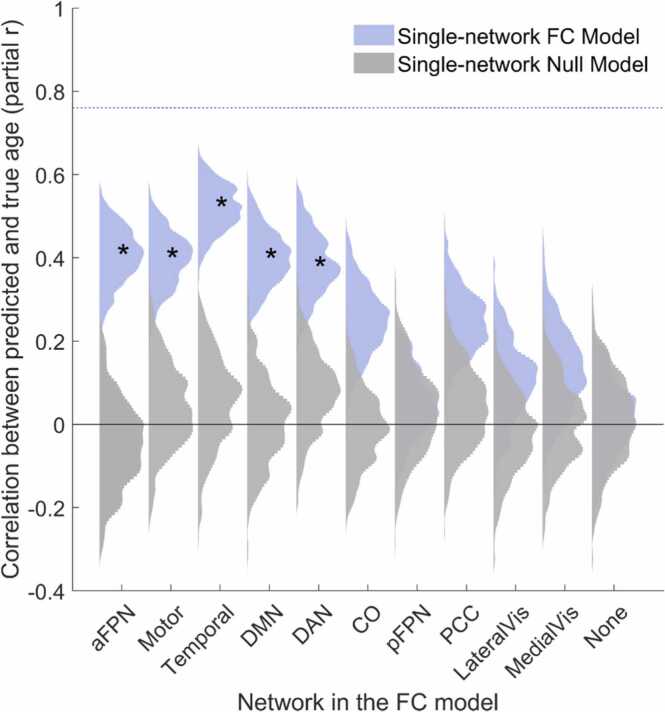
Predictive power of individual infant/toddler functional networks against null models. The distribution of partial correlation coefficients between true and predicted age (adjusted for nuisance variables) is shown light blue and corresponding null model distributions are shown in grey. *Indicates *p* < .05 for each network (comparison of true model’s median of partial r with the null distribution from 500 bootstraps) with Holm-Bonferroni correction for multiple comparisons. Networks are ordered by size, with the largest network (aFPN) on the left. The dashed blue line shows the *median* partial *r* of full within-network model from the analyses in Fig. 5 for comparison.

To assess the predictive power of each infant/toddler functional network, we trained separate SVR models using features from each of the 11 networks separately. Networks involving temporal regions, regions overlapping with the canonical dorsal attention network (DAN), motor areas, anterior fronto-parietal network (aFPN), and frontal parts of the default mode network (infant-DMN) all reliably changed over 8–26 months in manner that facilitates age prediction (Fig. 6). Networks involving the visual areas, on the other hand, did not predict brain maturation on their own better than chance in this age range.

We also measured the relationship of age with properties of single connections, namely in terms of an edge’s test-retest reliability (ICC) as well as its differential power (DP; the likelihood of a connection being more similar across runs of the same participant compared to other participants). These results are reported across all the 55,278 connections in in *Supplementary section 5*.

## 4 Discussion

In this study we found that 8-to-26-month-olds' resting-state functional connectivity patterns are relatively reliable within-individuals during the same scan session, distinct across individuals, and diagnostic of age in months. The first two findings extend prior work assessing the utility of preterm-to-early-infancy rsFC for characterizing functional brain architecture (Smyser et al., 2010, Gao et al., 2013, Rudolph et al., 2018) as well as recent investigations of rsFC reliability and individuality in neonates and younger infants (Dufford et al., 2021). The third finding complements previous works on predictive models of brain maturation in the first year of life (Pruett et al., 2015) and in later childhood, adolescence, and adulthood (Nielsen et al., 2019, Dosenbach et al., 2010).

Understanding the development of the brain’s functional architecture and its individual variability in very young ages may be useful for understanding both typical and atypical neurodevelopment. For rsFC to be a useful measure of neural development in infancy, however, it must be reliable: FC patterns that are not reproducible from one minute to the next are unlikely to reflect developmental change in functional brain organization. To predict developmental outcomes, FC patterns must also be, to some degree, distinct between individuals: if all infants showed an identical rsFC pattern this pattern would not be useful for distinguishing differences between them. To assess the stability and uniqueness of rsFC in our sample, we compared connectivity patterns calculated from two approximately 4-minute splits of fMRI data after rigorous motion exclusion and quality control. We found relatively stable FC multivariate patterns (split-half *r* = 0.5), which aligns with the medium intra-session reliability of rsFC in newborn infants reported by (Wang et al., 2021a). Single-edge ICC were low, however, which follows previous studies (Noble et al., 2019, Dufford et al., 2021). We also achieved successful functional connectome fingerprinting (Miranda-Dominguez et al., 2014, Finn et al., 2015), with 63% accuracy in identifying an individual from other infants and toddlers (with chance being ≈ 1/158). This individual reliability and distinctness in our sample of 112 8-to-26-month-olds is comparable to the fingerprinting identification accuracies reported for the first year of life (Dufford et al., 2021: *n* = 55 scanned at both 1 month and 9 months of age; best-case within-session ID accuracy = 49–71%) but lower than those reported by (Wang et al., 2021b) (*n* = 40 neonates ID accuracy = 100%).

Our results encourage examinations of *large-scale* functional brain network development in infancy. This is because, in addition to identifying individuals, whole-cortex functional connectivity patterns predict infants’ and toddlers’ age-in-months with relatively large prediction R^2^ (0.51) and a median prediction error of 3.6 months. Extending previous work predicting age from EEG functional connectivity in preterm and full-term neonates (Lavanga et al., 2018) and classifying 6- vs. 12-month-old fMRI functional connectivity patterns (Pruett et al., 2015), this finding demonstrates that rsFC patterns show rapid, stereotyped changes with age in early development. Furthermore, although a direct comparison of predictive power is difficult due to differences in age and brain measure variance, the current model performs nearly as well as connectome-based models of age in older children and adults defined with similar machine learning methods. For example, a recent model predicting age from rsFC in a sample of 7-to-35-year-olds achieved a prediction R^2^ of.57 (compared to.51 in the current sample) (Nielsen et al., 2019).

Do all infant-to-toddler functional connections change similarly with age? From a broad perspective, we observed significant differences in the degree to which functional connections *within* and *between* resting-state networks matured with age. Specifically, functional connections within infant-defined functional networks better predicted age (prediction R^2^ =.58) than functional connections crossing network boundaries (prediction R^2^ =.48). Although both types of connections predict age above and beyond brain volume, models trained on within-network edges significantly outperform models trained on between-network edges (median difference in prediction R^2^ = 10.0%), despite many fewer model features (6017 vs 49,261 edges). One possibility is that within-network models outperform between-network models simply because within-network edges are on average, stronger (i.e., Pearson correlations of the within-network edges are larger than the between-network edges; see *Supplementary section 6*). However, we found that within-network models still numerically outperform models based on the 25% strongest between-network edges, suggesting that edge strength is not the sole driver of this effect (see *Supplementary section 6* and Fig. S7). Together these results suggest that, in this age range, developmental change in between-network edges is more idiosyncratic than developmental change in within-network edges, making it less predictive of age across infants and toddlers.

In addition to comparing within- and between-network connections, we also used predictive models based on functional connections in individual networks. This analysis suggested that connections in frontal and temporal lobes, as well as motor and dorsal attention network regions, contributed most to age prediction in the 8-to-26-month range. Connections in visual areas, on the other hand, were not reliably related to age. These results are in line with studies showing improved synchronization within both the dorsal attention and the default networks during the first two years of life (Gao et al., 2013). They also complement previous findings from infants younger than one year that the attention/default-mode and executive control networks start to mature later than the visual and sensorimotor networks (Gao et al., 2015a, Gao et al., 2015b), as we found attention/default-mode networks are changing more (i.e., better predict age) in *later* infancy and in the *second* year of life compared to visual networks (Fig. 6). Notably, comparing the single-network results with the full within-network models shows that the combination of all 11 functional networks better predict age than any one network alone (see Fig. 6 dashed blue line). Additionally, a supplementary analysis (*Supplementary section 7*) showed that no single network outperforms size-matched models trained to predict age using random set of edges from outside of that network. Thus, single-network analyses reveal the networks that predict age above chance but demonstrate that no single network can outperform other connections outside of it to predict age.

The current results demonstrate that rsFC tracks neurodevelopment in infancy and toddlerhood. In addition, they may also have implications for connectome-based models of phenotypes beyond age in this age range. That is, resting-state FC has been used to predict phenotypes such as clinical diagnoses and cognitive abilities in typically and atypically developing adults and children (Smyser et al., 2016, Jahedi et al., 2017, Yamashita et al., 2018, Fair et al., 2013). Because it is challenging to characterize such phenotypes from behavior alone before language fluency and task competency develop, resting-state neural predictors of developmental outcomes could prove especially useful in infancy and toddlerhood (Fransson et al., 2011, Cusack et al., 2018, Linke et al., 2018, Marrus et al., 2018, Johnson et al., 2020). The relatively large and comparable-to-older-samples predictive power we found here indicates the tenability of using infant functional connectivity for predicting other phenotypic measures or individual differences in cognitive, language, or motor skills at very young ages. As long as the infant behavioral measures are reliable and reliably related to functional connectivity, their prediction with infant connectome-based predictive models could—with rigorous artifact and noise removal—be possible. For example, connectome-based predictive modeling of different behavioral measures has been successful in predicting outcomes such as ADHD symptoms or cognitive abilities among older children to adults samples (e.g., Rosenberg et al., 2016; Kardan et al., 2022), yet is currently underutilized in younger ages.

Studies aiming to relate infant rsFC to behavioral measures of development would benefit from our age-prediction model and understanding the signatures of growth in functional connectome during toddlerhood (Gao et al., 2020, Jasińska et al., 2021, Yu et al., 2021). For example, the difference between the predicted vs. observed chronological age in a participant may be related to their behavioral performance and cognitive development status (e.g., see Rudolph et al., 2017). Furthermore, assuming brain maturation causes the most profound changes in rsFC in infants and toddlers, our results could inform studies looking to predict individual differences in measures of cognitive development in this age range by providing a theoretical ceiling in predictive power for their rsFC-phenotype (brain-behavior) models. Additionally, we found that within-network connections are better predictors of maturation. Future work and studies aiming to predict individual differences in cognitive and other developmental outcomes in early development can determine whether there will be benefits from such dimensionality reduction in the functional connectivity space for their purposes or if inter-network relationships are important in those domains.

There are a few limitations to our study. First, although our sample size is relatively large in the field of infant fMRI, it is smaller in magnitude than recent sample sizes recommended for brain-based predictive models (e.g., Marek et al., 2020). Thus, it will be important for future work to externally validate the current age-prediction models across independent datasets to assess its generalizability. Second, because we found that infant FC predicts chronological age, indicative of brain maturity, in typical early development, our results may have clinical utility for the diagnosis of babies with atypical developmental trajectories (e.g., see Ciarrusta et al., 2020; Emerson et al., 2017). However, these models’ utility for predicting outcomes other than chronological age is untested. Third, despite using age-appropriate functional networks, the functional parcels we used to down-sample the voxel space were originally defined in adult resting-state data (Gordon et al., 2016). Infant-specific parcels may allow better network definitions or even higher age prediction accuracy. Finally, the functional connectome fingerprinting accuracy we found in our sample is lower than that observed in adult data. Despite the fact that more reliable FC data and better fingerprinting accuracy (within the same session) do not always correspond to better behavioral prediction (Noble et al., 2017, Finn and Rosenberg, 2021), studies characterizing developmental change in functional brain architecture should consider changes in functional connectome consistency and distinctiveness across infancy and toddlerhood. Despite these limitations, our findings contribute to a growing body of work characterizing FC reliability, distinctiveness, and predictive power in infancy and toddlerhood.

In conclusion, resting-state functional connectivity in infancy and toddlerhood is relatively stable within scan sessions, distinct across individuals, and informative of chronological age above and beyond brain volume. Connections within functional networks significantly outperform connections between networks when predicting age. Looking ahead, the current findings can help characterize changes in functional brain organization across early development and may inform work predicting phenotypes beyond age in infancy.

## Declaration of Competing Interest

The authors declare that they have no known competing financial interests or personal relationships that could have appeared to influence the work reported in this paper.

## Data Availability

The BCP data can be downloaded from the National Institute of Mental Health Data Archive (NDA) at https://nda.nih.gov/edit_collection.html?id= 2848. The preprocessing scripts are available at https://github.com/DCAN-Labs/dcan-infant-pipeline. The scripts used to generate the results in this study from the preprocessed data are available on https://github.com/okardan/BCP_Reliability_ID_Age .

## Footnotes

1 Accuracy for 2 * 300 frames is 70.7% for AP-to-PA and 72.0% PA-to-AP halves, separately. Accuracy values shown here (e.g., 62.0% for 2 ×300 frames) are based on when split-half FCs of a participant are more correlated than those of *both* AP and PA halves of other participants in the sample.
