# Supplementary material for: Resting-state functional connectivity identifies individuals and predicts age in 8-to-26-month-olds
Source: Dev Cogn Neurosci. 2022 Jun 15;56:101123. doi: 10.1016/j.dcn.2022.101123 (PMC9234342; doi:10.1016/j.dcn.2022.101123)
Supplement: Supplementary file 1 — Supplementary material [file mmc1.docx]

**Resting-state functional connectivity identifies individuals and predicts age in 8-to-26-month-olds**

Omid Kardan^1*^, Sydney Kaplan^2^, Muriah D. Wheelock^2^, Eric Feczko^3^, Trevor K. M. Day^3^, Óscar Miranda-Domínguez^3^, Dominique Meyer^2^, Adam T. Eggebrecht^2^, Lucille A. Moore^4^, Sooyeon Sung^3^, Taylor A. Chamberlain^1^, Eric Earl^4^, Kathy Snider^4^, Alice Graham^4^, Marc G. Berman^1^, Kamil Uğurbil^3^, Essa Yacoub^3^, Jed T. Elison^3^, Christopher D. Smyser^2^, Damien A. Fair^3^, Monica D. Rosenberg^1*^

^1^University of Chicago

^2^Washington University in St. Louis School of Medicine

^3^University of Minnesota

^4^Oregon Health & Science University

*Correspondence to [okardan@uchicago.edu](mailto:okardan@uchicago.edu) and [mdrosenberg@uchicago.edu](mailto:mdrosenberg@uchicago.edu)

**Supplementary Material**

*1. Examples of the AP and PA FC matrices from different subjects*


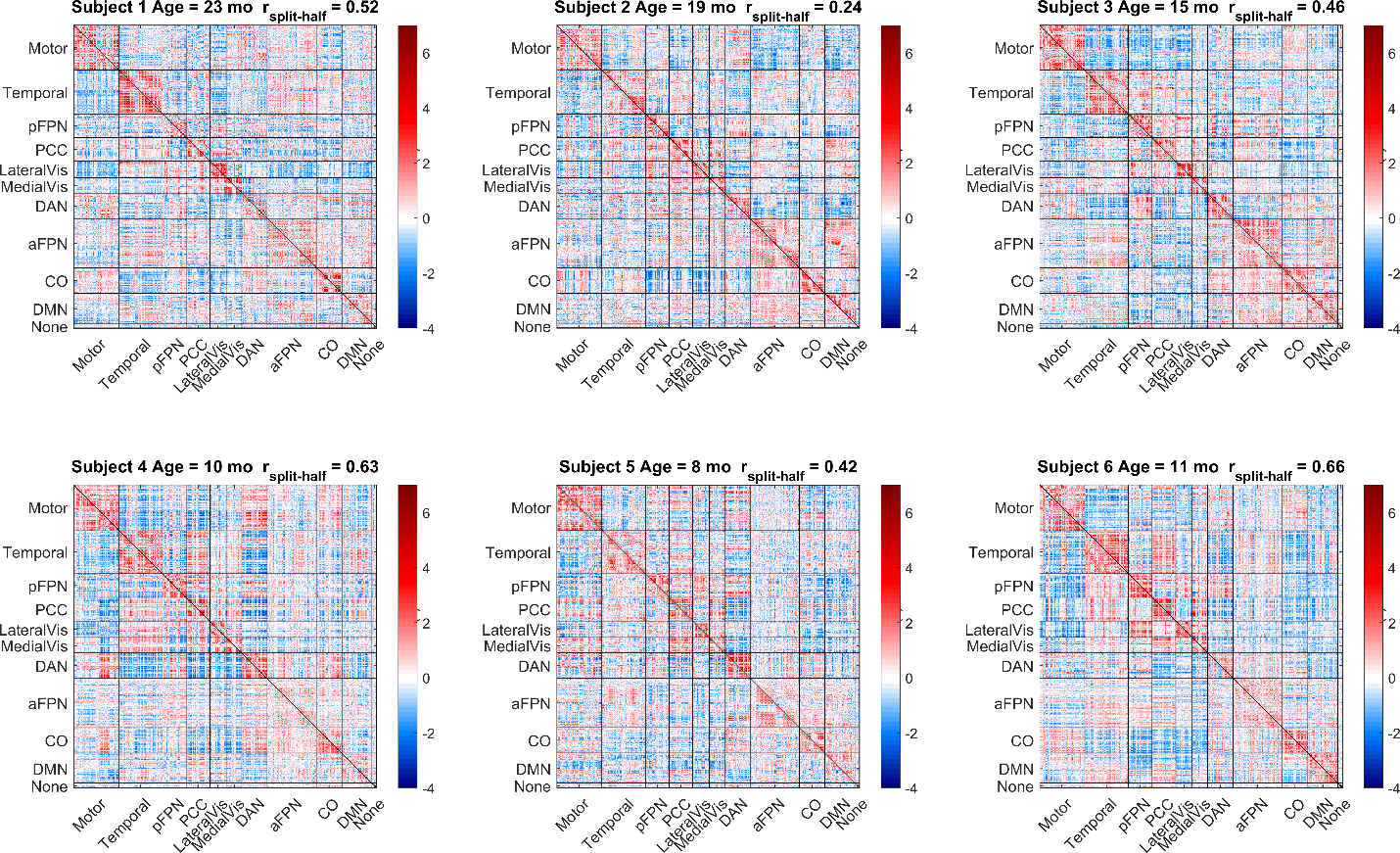


**Figure S1.** Functional connectivity matrices from the AP and PA halves of sessions presented below and above the main diagonal, respectively, in each plot. Each matrix plot shows a unique individual. The six subjects were selected pseudo-randomly (random but from different age ranges). The colorbars show the Fisher *z* transformed values of the connectivity in each FC.

*2. Support Vector Regression for age prediction*

The MATLAB 2018b function *fitrsvm* was used to fit and cross-validate SVR models. In SVR, a margin (a tube of width epsilon around the regression line in multivariate space) is defined where any data point (i.e., participants, or observations) within this tube incurs zero cost penalty. A penalty (loss) is assigned to data that lie too far from the regression line in multivariate space. On the other hand, the C parameter determines how strongly observations beyond the epsilon-insensitive tube are penalized, therefore controlling the trade-off between the slope of the regression line and cost assigned to poorer fit (larger C allows the regression line to be less flat, see Christianini & Shawe-Taylor, 2002). To be consistent with previous work predicting brain maturation using functional-connectivity-based SVR models (Dosenbach et al., 2010, Nielsen et al., 2019), we set the regularization parameters ‘BoxConstraint’ (i.e., C) and ‘Epsilon’ to infinity and 0.00001 respectively.^[[1]](#footnote-1)^ Predictors were standardized (*z*-scored) within each cross-validation loop and other parameters of SVR were set at the default (‘Solver’ = SMO and ‘KernelScale’ = 1; see <https://www.mathworks.com-/help/stats/fitrsvm.html> for more details about default parameters).

In 10-fold cross-validation, participants are partitioned into ten 90%-10% subsets. The machine learning model is fit in the 90% training data and applied to the left-out 10% test subset in each partition to evaluate generalizability within the cohort. The predicted age values for the left-out 10% were aggregated across the 10 folds to make two measures of model performance: Pearson correlation between predicted and true age (*r*) and *prediction* R^2^ = $1- \frac{\sum_{i=1}^{n} {(y_{i}-\hat{y}_{i})}^{2}}{\sum_{i=1}^{n} {(y_{i}-\bar{y})}^{2}}$ , where $y_{i}$ and $\hat{y}_{i}$ are the $i_{th}$ observed and predicted values, respectively, and $\bar{y}$ is the average of observed values. Pearson *r* is a commonly used measure for assessing model performance and reflects successful rank-discrimination in held-out participants. Prediction R^2^, a complementary measure, assesses numerical accuracy as it directly compares mean squared error (MSE) of the predicted and observed age values in the left-out folds, rather than the MSE of the predicted values and that of *a regression line* on observed ages in the left-out folds (i.e., *r*^2^) (Alexander, Tropsha, & Winkler, 2015). Prediction R^2^ can be negative if model’s error is worse than simply guessing the mean of the left-out observations, which can happen even when discriminating the rank order of the left-out participants is successful (i.e., *r* is significantly greater than 0).

*3. Gordon 2016 adult and BCP-based infant networks*


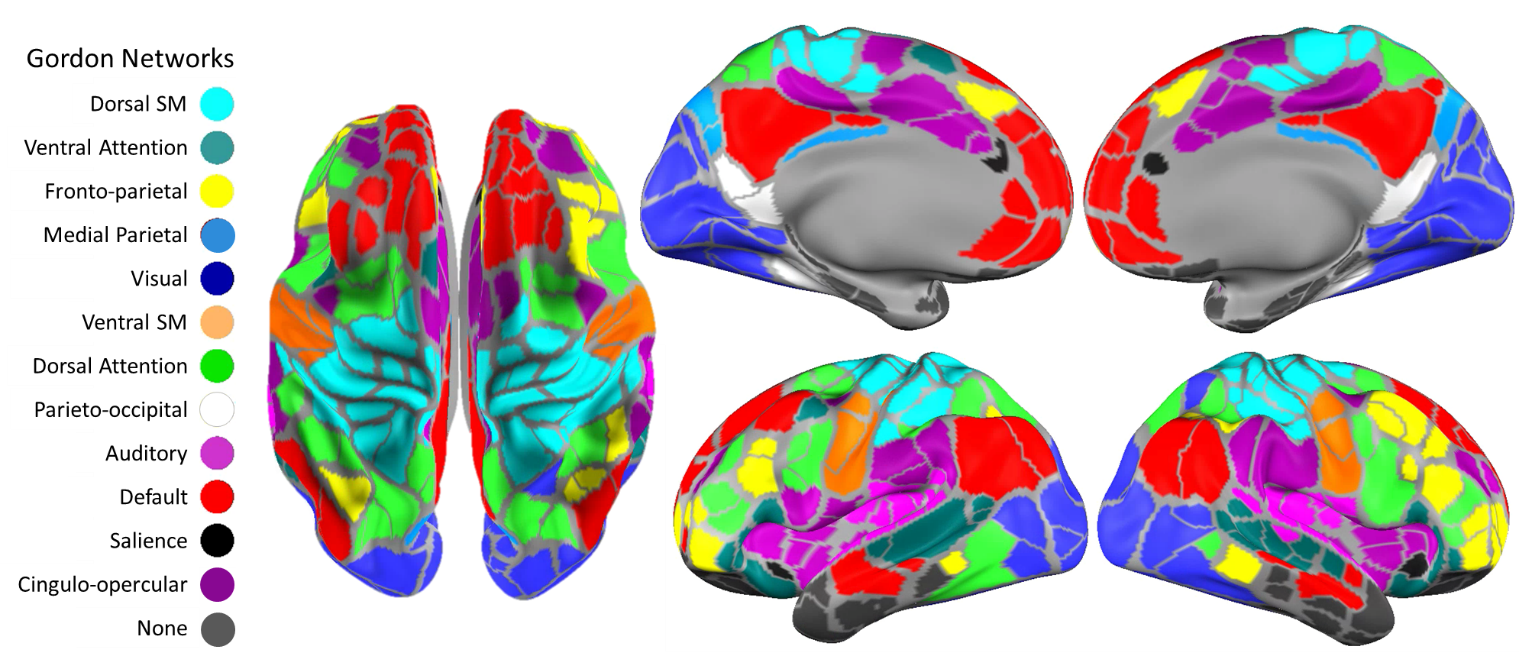


**
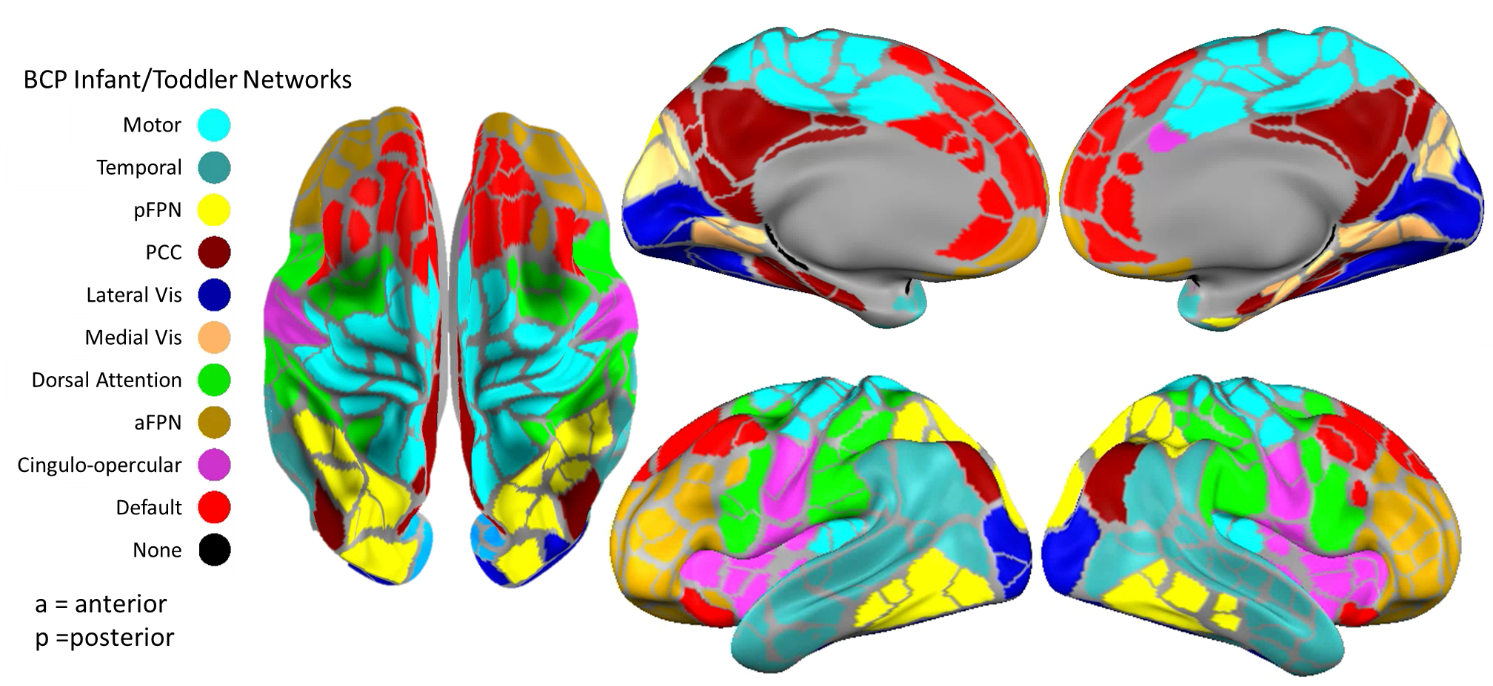
**

**Figure S2.** Gordon et al. (2016) adult functional network assignments (top) and the BCP-based infant and toddler functional network assignments (bottom). Node boundaries are identical in both cases; only the nodes’ network assignments differ.

*4. Within-network connections and between-network connections predicting age*


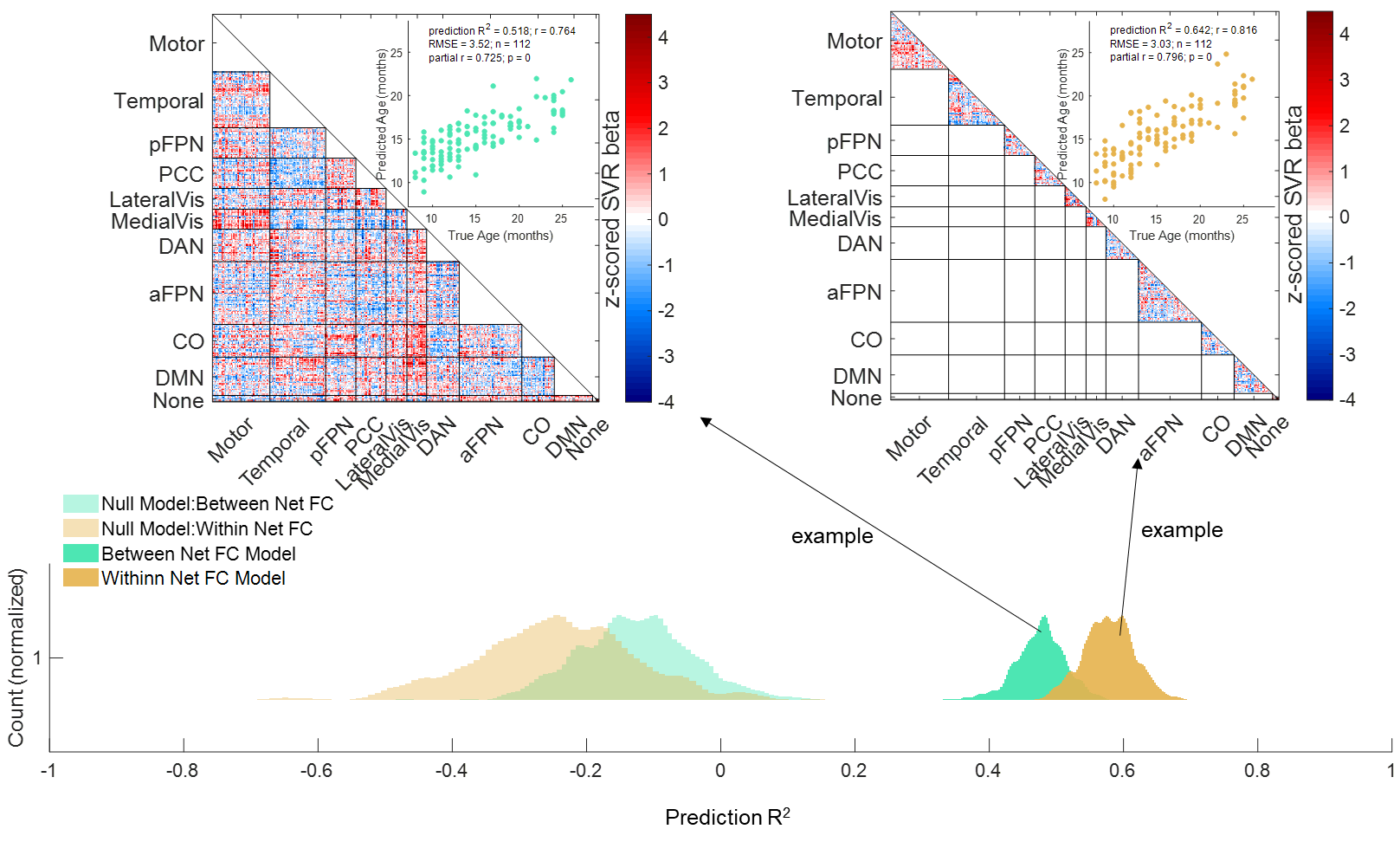


**Figure S3.** Performance of rsFC age-prediction models using only within-network connections (orange) or between-network connections (teal). Model feature weights (SVR beta weights) from one example SVR in each of the distributions is shown with arrows, with a corresponding scatterplot of predicted versus true age above the diagonal of the feature weights matrix.

*5. Relationship of ICC and DP of edges with their contribution to age prediction*

There was no relationship between the test-retest reliability (ICC) of edges and their beta coefficient from the SVR age-prediction models (mean r = -.019, SD = .018 across 500 boostrapped SVR models).

We also measured the differential power (DP) of each edge in the connectomes by calculating the likelihood of each edge being more similar from two runs of the same subject compared to unmatched runs (Finn et al 2015). DP for edge $e$ of subject $i$ was calculated as:

$$DP\left( i, e \right)-ln\frac{({|r\left( e \right)}_{ii}<{r\left( e \right)}_{ij}\left| +{|r\left( e \right)}_{ii}<{r\left( e \right)}_{ji} \right|)}{2(n-1)}$$

Where $e$ = 1, 2, .., 55278 (edges); $i and j$ = 1, 2, 112 (subjects), and $n$ = 112. ${r\left( e \right)}_{ii}$ is the product of functional connectivity value for edge $e$ in the two runs of subject $i$ while ${r\left( e \right)}_{ij}$ is the product of functional connectivity value for edge $e$ in the two runs of non-matching subjects By summing up $DP\left( i, e \right)$ across participants, we get $DP\left( e \right)$. If the within-participant product is higher than between-participant product across all the participants in the sample, this edge may be contributing more to the identification analysis (connectome fingerprinting). The DP values for all edges are presented in Figure S4 below the diagonal.

**
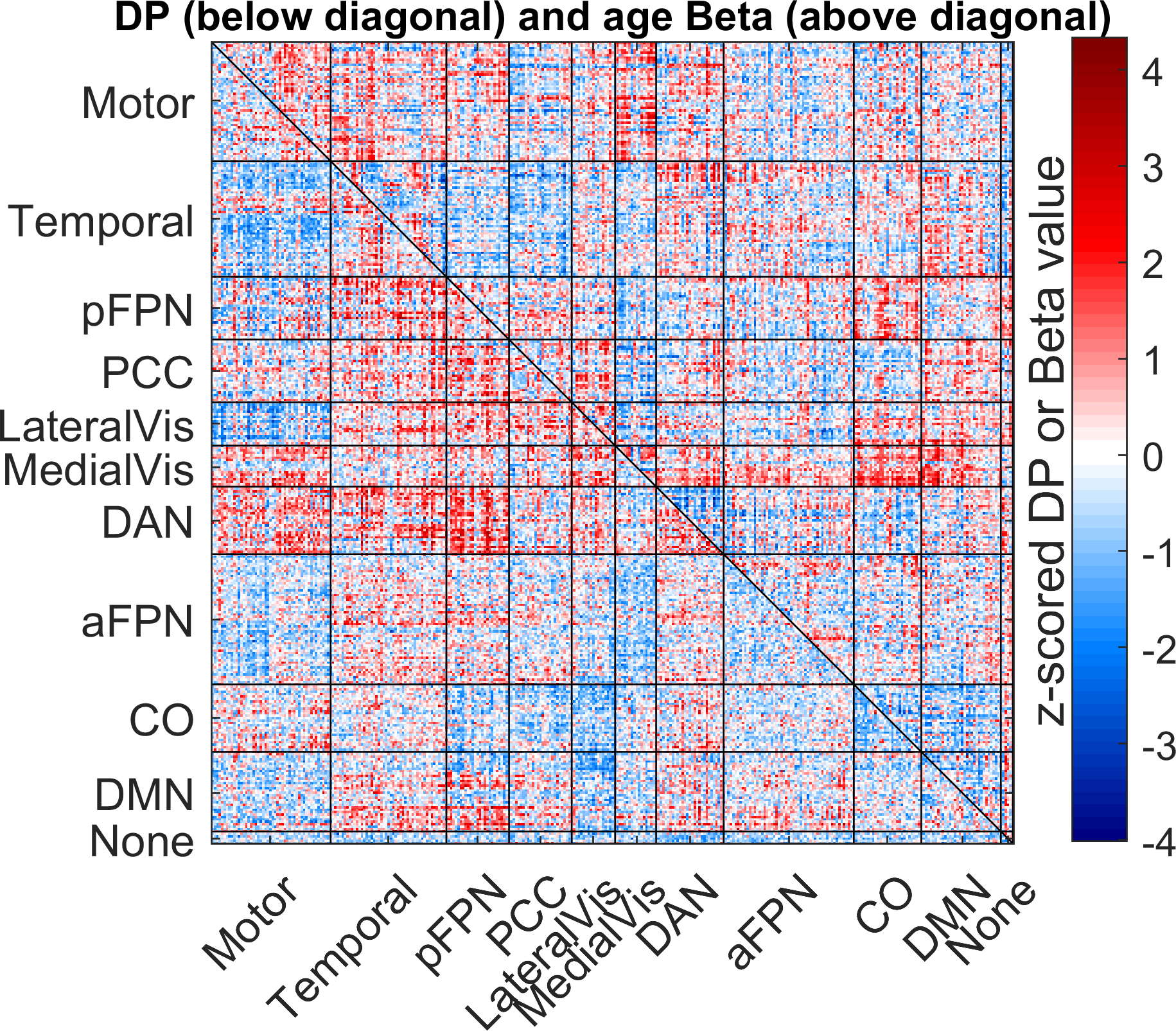
**

**Figure S4.** Differential power (below the diagonal) and the age-prediction SVR beta values (above the diagonal) are shown for all the edges in the functional connectome. Lack of diagonal symmetry (over the main diagonal) suggests no apparent relationship between the DP and age-prediction contribution across the edges.

The DP values had a very small but negative correlation with age prediction beta coefficients across edges (*r* = -.037, p <1/500, SD = .015), showing that edges with higher DP did not contribute more to predicting age in our sample (Figure S3).

*6. Within-network connections numerically outperform strong between-network connections for predicting age*

Because within-network edges were more strongly positive (mean *r* = .261, SD = .165) than between-network connections (mean *r* = -.003, SD = .112), we performed a control analysis to ask whether they better-predict age *only* because they are strong functional connections or because they relate to maturation beyond simply being more reliable and less noisy. To do so, we used the top 6017 edges (top ~12%, mean *r* = .209, SD = .081) strongest positive edges from the 49,261 between-network connections across the sample 500 times and trained SVR models to predict age using these connections. Within-network FC models numerically outperformed these strong edges and feature-size-matched models (*p* = .066, ΔR^2^ = .043; see supplementary **Figure S5**), suggesting that edge strength may not be the only factor to account for the power of within-network edges for predicting age in infants and toddlers.


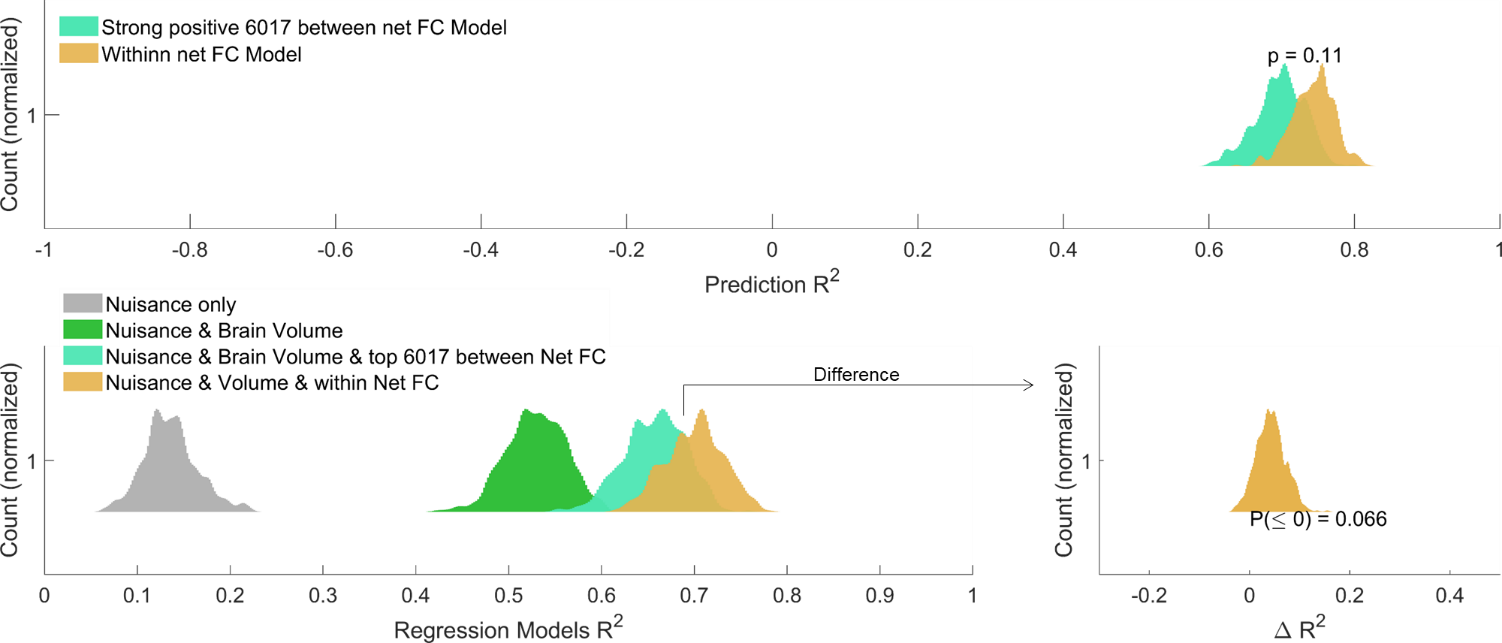


**Figure S5.** Models trained with 6017 (top 12%) strongest positive edges from the between-network set of connections still predict age worse than the within-network FC model.

*7. Single-network models cannot predict age better than control models trained to predict age using same number of edges from outside of the corresponding network*


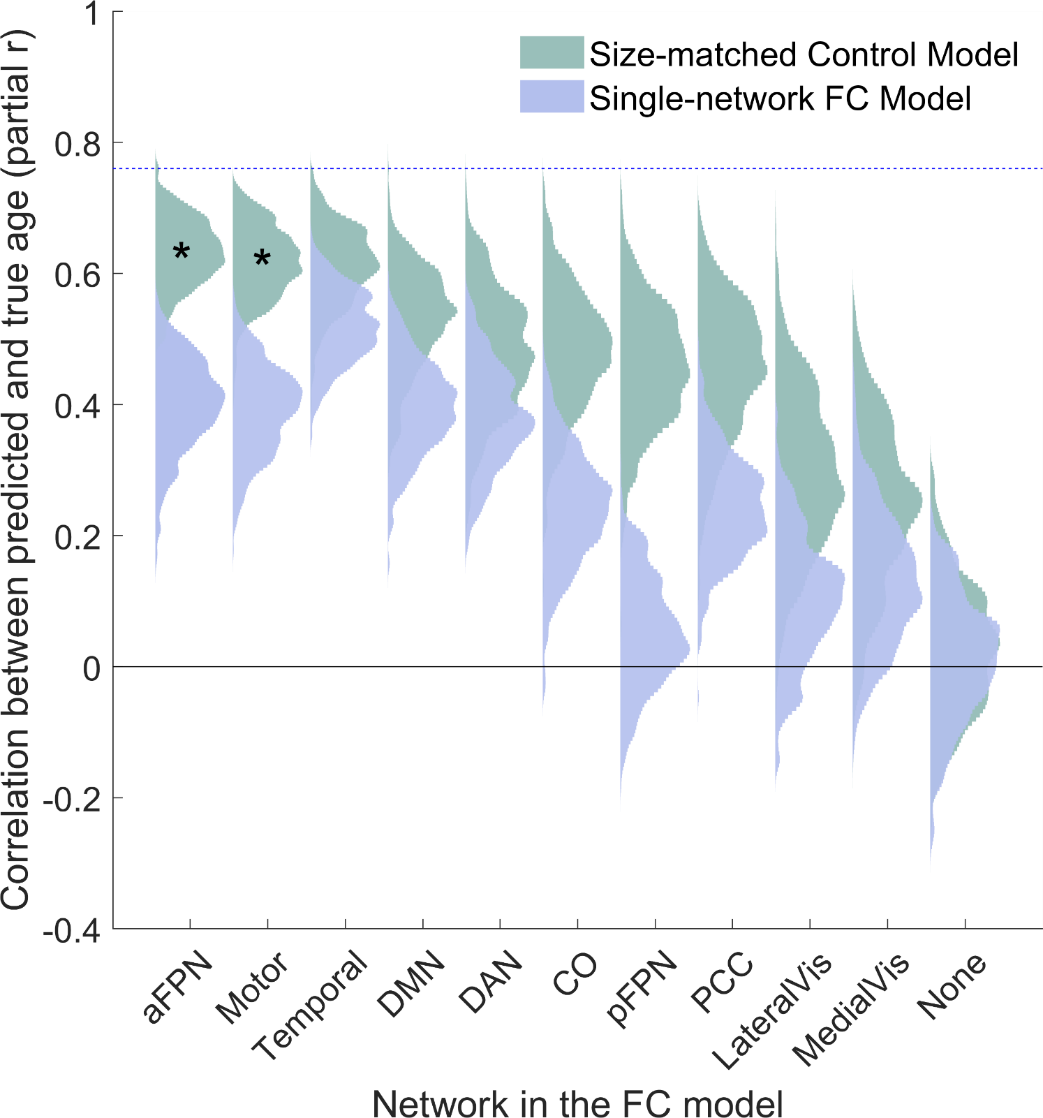


**Figure S6.** Predictive power of individual infant/toddler functional networks against control models with random edges from outside that network. The distribution of partial correlation coefficients between true and predicted age (adjusted for nuisance variables) is shown light blue and corresponding control model distributions are shown in green. *Indicates *p* < .05 for each pair of distributions. Networks are ordered by size, with the largest network (aFPN) on the left. The dashed blue line shows the *median* partial *r* of full within-network model from the analyses in Figure 5.

1. Notably, changing these parameters to the default *fitrsvm* function values of 1 and iqr(Y)/13.49, where iqr(Y) is the interquartile interval and Y is the subject ages, made almost no difference in the prediction R^2^ values. [↑](#footnote-ref-1)
